# Genomic and *in Situ* Analyses Reveal the *Micropruina* spp. as Abundant Fermentative Glycogen Accumulating Organisms in Enhanced Biological Phosphorus Removal Systems

**DOI:** 10.3389/fmicb.2018.01004

**Published:** 2018-05-23

**Authors:** Simon J. McIlroy, Cristobal A. Onetto, Bianca McIlroy, Florian-Alexander Herbst, Morten S. Dueholm, Rasmus H. Kirkegaard, Eustace Fernando, Søren M. Karst, Marta Nierychlo, Jannie M. Kristensen, Kathryn L. Eales, Paul R. Grbin, Reinhard Wimmer, Per Halkjær Nielsen

**Affiliations:** ^1^Center for Microbial Communities, Department of Chemistry and Bioscience, Aalborg University, Aalborg, Denmark; ^2^School of Agriculture, Food, and Wine, The University of Adelaide, Adelaide, SA, Australia

**Keywords:** activated sludge, EPBR, fermentation, *Micropruina*, GAO, PAO

## Abstract

Enhanced biological phosphorus removal (EBPR) involves the cycling of biomass through carbon-rich (feast) and carbon-deficient (famine) conditions, promoting the activity of polyphosphate accumulating organisms (PAOs). However, several alternate metabolic strategies, without polyphosphate storage, are possessed by other organisms, which can compete with the PAO for carbon at the potential expense of EBPR efficiency. The most studied are the glycogen accumulating organisms (GAOs), which utilize aerobically stored glycogen to energize anaerobic substrate uptake and storage. In full-scale systems the *Micropruina* spp. are among the most abundant of the proposed GAO, yet little is known about their ecophysiology. In the current study, genomic and metabolomic studies were performed on *Micropruina glycogenica* str. Lg2^T^ and compared to the *in situ* physiology of members of the genus in EBPR plants using state-of-the-art single cell techniques. The *Micropruina* spp. were observed to take up carbon, including sugars and amino acids, under anaerobic conditions, which were partly fermented to lactic acid, acetate, propionate, and ethanol, and partly stored as glycogen for potential aerobic use. Fermentation was not directly demonstrated for the abundant members of the genus *in situ*, but was strongly supported by the confirmation of anaerobic uptake of carbon and glycogen storage in the absence of detectable polyhydroxyalkanoates or polyphosphate reserves. This physiology is markedly different from the classical GAO model. The amount of carbon stored by fermentative organisms has potentially important implications for phosphorus removal – as they compete for substrates with the *Tetrasphaera* PAO and stored carbon is not made available to the “*Candidatus* Accumulibacter” PAO under anaerobic conditions. This study shows that the current models of the competition between PAO and GAO are too simplistic and may need to be revised to take into account the impact of potential carbon storage by fermentative organisms.

## Introduction

Enhanced biological phosphorus removal (EBPR) activated sludge systems have been widely implemented for the removal of nutrients from wastewaters. Phosphorus (P) removal is achieved in these systems by cycling of the biomass through carbon-rich (feast) anaerobic and carbon-deficient (famine) conditions to encourage the activity of the so-called polyphosphate accumulating organisms (PAOs) [see Oehmen et al. (2007) for review].

Classical models for the PAO phenotype stipulate that aerobically stored polyphosphate provides energy for anaerobic uptake and storage of volatile fatty acids (VFAs) as polyhydroxyalkanoates (PHAs). Hydrolysis of aerobically stored glycogen, and activity of the tricarboxylic acid (TCA) cycle provides required reducing power and additional energy. Stored PHAs are utilized under subsequent aerobic conditions supporting growth and replenishing glycogen and polyphosphate stores, with wastage of aerobic biomass giving net P removal (Comeau et al., 1986; Wentzel et al., 1986: Mino et al., 1987). Such a phenotype has been demonstrated in the widely studied “*Ca.* Accumulibacter” genus within the Betaproteobacteria (Hesselmann et al., 1999; He and McMahon, 2011).

Although EBPR systems are considered as an economical strategy for wastewater treatment, they are subject to periods of inefficiency and failure. Deterioration of EBPR has been attributed to a variety of conditions; such as high rainfall, nutrient limitation, and high nitrate loading to the anaerobic zone (Oehmen et al., 2007). Another proposed reason is microbial competition, where the proliferation of organisms competing for anaerobic carbon supply, without excess polyphosphate storage, is at the theoretical expense of P removal efficiency (Satoh et al., 1994). Bacteria with the glycogen accumulating organism (GAO) phenotype have received considerable attention as potential competitors of the PAO (Oehmen et al., 2007). The GAO phenotype is similar to the classical PAO phenotype, except that polyphosphate is not stored in excess under aerobic conditions. As polyphosphate is not stored for anaerobic use, there is an increased reliance on aerobically stored glycogen as an energy source for anaerobic carbon uptake (Liu et al., 1994; Mino et al., 1995). The phenotype has been demonstrated for members of the alphaproteobacterial *Defluviicoccus* (Wong et al., 2004), and the gammaproteobacterial Competibacteraceae family (Crocetti et al., 2002; Kong et al., 2002; McIlroy et al., 2015a) and at least partially for several other taxa (see Stokholm-Bjerregaard et al., 2017).

Research efforts have overwhelmingly focused on the competing classical PAO–GAO phenotypes in EBPR with acetate as carbon source. It is generally believed that VFAs are made available to the PAO and GAO through the hydrolysis and fermentation of more complex substrates, such as carbohydrates and proteinaceous material, by other heterotrophic organisms (Henze et al., 2000; Kong et al., 2008). However, more diverse strategies for organisms with a direct influence on EBPR have long been suggested (Carucci et al., 1999). Several anaerobic carbon storage compounds are reported for full-scale activated sludge, including triacylglycerols (TAGs), gamma-aminobutyric acid (GABA) as well as intracellular pools of non-polymerized fermentation by-products, long chain fatty acids, amino acids, and trehalose (Satoh et al., 1998; Santos et al., 1999; Kristiansen et al., 2013; McIlroy et al., 2013; Nguyen et al., 2015; Marques et al., 2017). Some organisms exhibiting the classical GAO and PAO phenotypes reportedly utilize sugars and amino acids directly for PHA production (Liu et al., 1996; Burow et al., 2007; Oyserman et al., 2015) and some may also ferment glycogen stores or glucose to lactate as an additional anaerobic energy source (McIlroy et al., 2014). Most notably, the *Tetrasphaera* spp. and *Microlunatus phosphovorus*^T^, both within the phylum Actinobacteria, cycle polyphosphate without PHA storage with dynamic feast–famine conditions. Instead, these organisms exhibit a fermentative metabolism where polyphosphate supplements anaerobic energy demands (Nakamura et al., 1995; Kong et al., 2005; Kristiansen et al., 2013). Importantly, surveys of full-scale systems revealed that the *Tetrasphaera* spp. are in much higher abundance than the “*Ca.* Accumulibacter” PAO, questioning the long-held belief that the latter classical-PAO are the most important for EBPR (Nguyen et al., 2011; Mielczarek et al., 2013a; Saunders et al., 2016) and reinforcing the need to consider diverse physiologies for organisms important for EBPR.

In addition to the fermentative actinobacterial PAO, it has been shown that in dynamic feast–famine systems unidentified organisms can store glucose directly as glycogen anaerobically, energized by fermentation, without cycling polyphosphate (Carucci et al., 1999). The role of these “fermentative GAO” in EBPR is of interest, given their potential as competitors of the abundant fermentative *Tetrasphaera* PAO. A likely candidate for this phenotype is the activated sludge isolate *Micropruina glycogenica*^T^ – a member of the family Propionibacteriaceae within the Actinobacteria – shown to accumulate large amounts of unidentified intracellular carbohydrate (up to 8.4% dry cell weight), in the absence of detected polyphosphate storage, under both aerobic and anaerobic conditions (Shintani et al., 2000). Members of the *Micropruina* genus were reportedly abundant in a non-EBPR lab-scale system fed with acetate and glucose (Kong et al., 2001). *In situ* analyses of the genus revealed that both acetate and glucose were taken up anaerobically with some PHA storage. A relatively high biomass polymerized-glucose content was attributed to storage by the *Micropruina* spp. present. The organism has received little attention in EBPR research since. However, in our recent comprehensive survey of full-scale EBPR plants in Denmark, the genus was observed to be the most abundant of the putative GAO proposed thus far (Stokholm-Bjerregaard et al., 2017). In the current study, several approaches were applied to the characterization of members of the *Micropruina* genus, in both pure culture and *in situ*, to give a comprehensive view of their ecology in EBPR systems and their subsequent importance therein. The results show that the classical view of PAO–GAO interactions in full-scale EBPR systems needs to be revised.

## Materials and Methods

### Pure Culture Studies

*Micropruina glycogenica* str. Lg2^T^ (DSM15918) was obtained from DSMZ and maintained on R2A agar (Reasoner and Geldreich, 1985) at 30°C. Anaerobic cultivation was performed in quadruplicate by autoclaving 40 ml R2A broth, without soluble starch, in serum flasks closed with rubber plugs perforated by syringe needles. Afterward, 0.2 μm filters were attached to the needles and nitrogen was bubbled through the hot media until it reached room temperature. A *Micropruina* colony was suspended in 200 μl R2A broth and distributed as inoculum to the nitrogen-saturated serum flasks. These were incubated for 7 days at 30°C.

### Nuclear Magnetic Resonance (NMR) Spectroscopy

Following incubation, cells were removed by centrifugation (10,000 ×*g*, 10 min, 4°C). For NMR analysis two approaches were chosen: with and without a concentration step. Samples without concentration were prepared by adding 60 μl of buffer [for 50 ml: 10 ml D_2_O, 0.04 g sodium 2,2,3,3-tetradeutero-3-trimethylsilyl propionate (TSP-d_4_), 1.005 g Na_2_HPO_4_ × 7 H_2_O, 0.5 ml NaN_3_ (4%), adjusted to 50 ml and pH 7.0 with H_2_O, and NaOH/HCl] directly to 540 μl of the cultures supernatant or the R2A control. For samples with a concentration step, 15 ml of the cultures supernatant or R2A was freeze-dried and suspended in 600 μl NMR sample solution [D_2_O containing 2 mM sodium azide and ∼0.83 mM trimethylsilylpropanoic acid (TSP)]. All samples were adjusted to a pH of 7.0 before NMR analysis.

All NMR spectra were recorded at 298.1 K on a BRUKER AVIII-600 MHz NMR spectrometer equipped with a 5 mm cryogenic inverse triple-resonance probe. A blank containing only water and buffer or the NMR sample solution was analyzed by a PULCON experiment and the exact TSP concentration was determined (Wider and Dreier, 2006). For each sample, a 1D-NOESY experiment with a relaxation delay of 1 s, a mixing time of 10 ms, and an acquisition time of 4 s were recorded. During the relaxation delay, the water resonance was presaturated with a continuous wave irradiation at γ*B*_1_/2π = 48 Hz. A pulsed field gradient of 1.2 ms length and 30 G/cm strength was applied between the presaturation period and the first 90° pulse. Another pulsed field gradient of 1.2 ms length and 7.2 G/cm was applied during the mixing period. Metabolite identification and quantification were performed using the ChenomX NMR Suite v. 8.1 using the known concentration of TSP as an internal standard.

### Genome Sequencing

Genomic DNA was extracted from *M. glycogenica* Lg2^T^ using a DNeasy Blood & Tissue Kit (Qiagen, Copenhagen, Denmark) according to manufacturer’s instruction with changes in the pre-treatment protocol for Gram-positive bacteria (2 h of lysis buffer treatment and 1 h of proteinase K treatment). A library for Illumina paired-end sequencing was prepared from 50 ng of total DNA using the Nextera DNA Library Preparation Kit (Illumina, San Diego, CA, United States) according to manufacturer’s instructions (Part No. 15027987 v01), but with an AMPure XP bead to sample ratio of 0.5:1 instead of 1.5:1 during the library clean-up step. The library was sequenced on the Illumina MiSeq platform (v3 chemistry, 2 × 300 bp). Paired-end reads were quality filtered (minimum length of 240 bp, allowing no ambiguous nucleotides) and trimmed for sequencing adaptors using the CLC genomics workbench (v. 9.5.2). In addition, long-read nanopore sequencing was performed. Genomic DNA was sequenced using the MinION (Oxford Nanopore Technologies, United Kingdom) following the manufacturer’s protocol (1D gDNA sequencing SQK-LSK108). The library was sequenced on a FLO-MIN106 flowcell on a MinION MK1b sequencer using Minknow software (v. 1.4.2). The reads were basecalled using Metrichor (v. 2.45.3). Fastq read files were extracted using Poretools [v.0.6.0 (Loman and Quinlan, 2014)]. The nanopore sequencing reads were assembled into a closed, single contig, draft genome using CANU [v. 1.4 (Koren et al., 2017)]. The draft genome was subsequently polished with the Illumina paired-end reads using Pilon [v. 1.18 (Walker et al., 2014)] and by manual read mapping in CLC genomics workbench to remove SNPs and ensure a high-quality assembly.

### FISH Probe Design and Optimization

Phylogenetic analyses and probe design were performed with the MiDAS database v. 2.1 (McIlroy et al., 2015b) [derived from the SILVA taxonomy (Quast et al., 2013)] within the ARB software package (Ludwig et al., 2004). Potential probes were assessed *in silico* with the mathFISH software for hybridization efficiencies of target, and potentially weak, non-target matches (Yilmaz et al., 2011). Unlabeled competitor probes were designed for single base mismatched non-target sequences (Manz et al., 1992). The Ribosomal Database Project (RDP) PROBE MATCH function (Cole et al., 2014) was used to screen for the existence of non-target indel sequences (McIlroy et al., 2011). Probe validation and optimization were based on generated formamide dissociation curves (Daims et al., 2005) where fluorescent intensities of at least 50 cells were measured with ImageJ software (National Institutes of Health, Bethesda, MD, United States). Calculated fluorescence intensity average values were compared for hybridization buffers with formamide concentrations over a range of 0–70% (v/v) with 5% increments (data not shown). *M. glycogenica* Lg2^T^ was used to optimize the MGL-67 and MGL-1223 probes designed to target the genus. Other isolates were used to assess the potential for non-specific binding of the designed probes (**Table 1**).

**Table 1:** FISH probes designed for the detection of members of the genus *Micropruina.*

Probe	*Escherichia coli* position	Target	Coverage^1^	Non-target hits^1^	Sequence 5′–3′	[FA] %^2^	Ref.
MGL-67	67–97	*Micropruina* spp.	8/9 (89%)	0	CAG AAG AGC AAG CTC TTC GTC ACC G	50	This study
MGL-67_C1**^3^**	67–97	Competitor probe for MGL-67	–	–	CAG AAG AGC AAG CTC TCC GTC ACC G	–	This study
MGL-1223	1223–1246	*Micropruina* spp.	8/9 (89%)	0	CCA GCC ATT GTA GCA TGT TTC AAG	40	This study
MGL-1223_C1**^3^**	1223–1246	Competitor probe for MGL-1223	–	–	CCT GCC ATT GTA GCA TGT TTC AAG	–	This study
MGL-1223_C2**^3^**	1223–1246	Competitor probe for MGL-1223	–	–	CCA GCC ATT GTA GCA TGT TTG CAG	–	This study
MIC184	645–661	*Micropruina* spp.	8/9 (89%)	22	CAT TCC TCA AGT CTG CC	20	Kong et al., 2001

**FIGURE 1:**
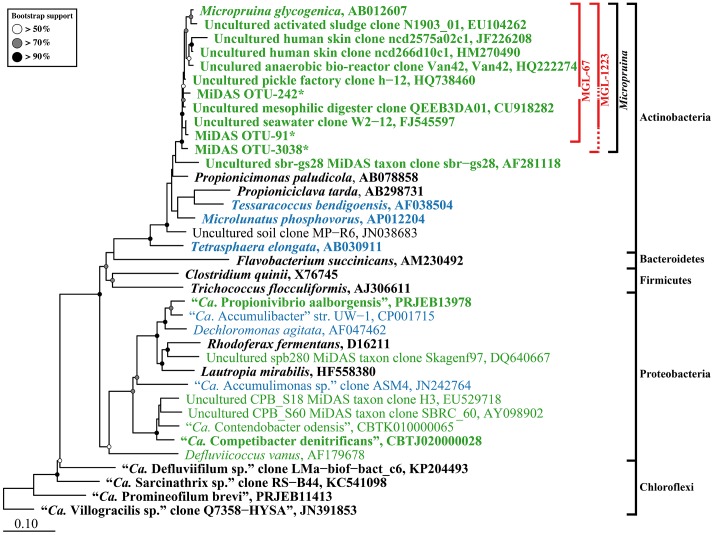
Maximum-likelihood (PhyML) 16S rRNA gene phylogenetic tree for PAOs, GAOs, and abundant fermenters in EBPR-activated sludge WWTPs. Sequences representing genera with known fermentative members are in bold typeface. Sequences representing putative PAO and GAO are blue and green, respectively. Outer brackets to the right indicate the phylogenetic classification of sequences. Inner red brackets show probe coverage; broken lines indicate the sequence information at the probe site is unavailable. The tree was prepared using the ARB software (Ludwig et al., 2004) from the MiDAS database (version 1.20), which is a version of the SILVA database [Release 119 NR99 (Quast et al., 2013)] curated for activated sludge organisms (McIlroy et al., 2015b). Sequences were aligned in the ARB software, trimmed, and variable regions excluded with a custom filter (filter by base frequency, 40–100%) leaving 1100 aligned positions. ^∗^Short MiDAS OTU amplicon sequences (461–462 bp) were added after calculation of the tree with the ARB insert sequences function. Bootstrap values from 100 analyses are indicated when above 50%. The scale bar represents substitutions per nucleotide base.

*^1^Coverage and specificity values are based on the MiDAS taxonomy v. 1.20 (McIlroy et al., 2015b) noting that the only sequence not covered by the three probes (Accession No. JN038683) does not consistently cluster with other sequences in the genus with phylogenetic analysis (**Figure 1**). ^2^Recommended hybridization buffer formamide concentration % [v/v]. ^3^Pure cultures representing non-target sequences with a single mismatch were used to assess the need for competitor probes. MGL-67_C1 and MGL-1223_C1 were required to remove non-specific binding of the MGL-67 and MGL-1223 probes to Propionicicella superfundia^T^ DSM22317 and Aestuariimicrobium kwangyangense^T^ DSM21549, respectively. Several database sequences contain two terminal mismatches to the MGL-1223 probe, which are covered by the MGL-1223_C2 competitor. Nonomuraea soli^T^ DSM45533 could not be used to assess the need for MGL-1223_C2 due to high autofluorescence. It is recommended that all listed competitor probes be applied with their respective probe.*

### Fluorescence *in Situ* Hybridization (FISH)

Fluorescence *in situ* hybridization was performed essentially as detailed by Daims et al. (2005). Activated sludge biomass samples from full-scale EBPR wastewater treatment plants (WWTPs) were taken from the aerobic tank and transported on ice to the laboratory as part of the broader MiDAS project (Mielczarek et al., 2013b). Activated sludge biomass and axenic cultures were fixed for FISH with 50% ethanol (v/v) and stored at -20°C. The 5′-end of oligonucleotide FISH probes were labeled with 5(6)-carboxyfluorescein-*N*-hydroxysuccinimide ester (FLUOS) or with the sulfoindocyanine dyes (Cy3 and Cy5) (Thermo Fisher Scientific GmbH, Ulm, Germany). The NON-EUB nonsense probe was used as a negative hybridization control (Wallner et al., 1993). Quantitative FISH (qFISH) values were calculated as a percentage area of the total biovolume, hybridizing the EUBmix probes (Amann et al., 1990; Daims et al., 1999), that also hybridized with the specific probe. qFISH analyses were based on 30 fields of view taken at 630× magnification using the daime image analysis software (DOME, Vienna, Austria) (Daims et al., 2006). Increased permeabilization of cells for FISH was achieved with the enzymatic pre-treatment method described by Kragelund et al. (2007). The protocol includes lysozyme treatment (Sigma–Aldrich, Brøndby, Denmark) (10 mg ml^-1^ in 0.05 M EDTA, 0.1 M Tris–HCl, pH 8) for 30 min at 37°C, achromopeptidase treatment (Sigma–Aldrich, Brøndby, Denmark) (60 U ml^-1^ in 0.01 M NaCl, 0.01 M Tris–HCl, pH 8) for 30 min at 37°C, and acid treatment with 0.1 M HCl for 10 min with a final dehydration step with 96% (v/v) ethanol for 1 min. Microscopy was performed with either an Axioskop epifluorescence microscope (Carl Zeiss, Oberkochen, Germany) or a white light laser confocal microscope (Leica TCS SP8 X).

### Microautoradiography–FISH

Samples were taken from the aerobic tank of the WWTPs in Odense North–West and Ejby Mølle, Denmark. Both plants are configured for EBPR with stable performance [see Mielczarek et al. (2013a) for further operational details]. Samples were stored at 4°C and microautoradiography (MAR) analyses performed within 24 h of sampling. The MAR protocol was essentially as detailed by Nierychlo et al. (2015). Activated sludge was aerated for 40 min at room temperature prior to MAR incubation, to reduce any residual substrates, oxygen, and NO*_x_* present. Sludge was then diluted with filtered sludge water from the same plant to yield a biomass concentration of 1 mgSS ml^-1^ for a final volume of 2 ml in 11 ml serum bottles. Radiolabeled substrates were added to yield a total radioactivity of 10 μCi mg^-1^ SS. The following were used: [^14^C]-pyruvic acid (PerkinElmer, Waltham, MA, United States), [^3^H]-acetic acid, [^3^H]-galactose, [^3^H]-oleic acid (Amersham Biosciences, United Kingdom), [^3^H]-glucose (PerkinElmer, Waltham, MA, United States), [^14^C]-propionic acid, [^3^H]-NAG, [^3^H]-fructose, [^14^C]-butyric acid, [^3^H]-glycerol, [^3^H]-ethanol, and [^3^H]-amino acid mix (American Radiolabeled Chemicals Inc., Saint Louis, MO, United States). The corresponding cold substrate was added to yield a total concentration of 2 mM. Oxygen was removed by repeated evacuation of the headspace and subsequent injection of O_2_-free N_2_ to achieve anaerobic conditions, prior to substrate addition. Samples were incubated with each labeled substrate for 3 h at room temperature (approx. 21°C) on a rotary shaker at 250 rpm. A pasteurized biomass (heated to 70°C for 10 min) was prepared as a negative control to assess possible silver grain formation due to chemography. Incubations were terminated by the addition of cold PFA to a final concentration of 4% (w/v). Samples were fixed for 3 h at 4°C and subsequently washed three times with sterile filtered tap water. Aliquots of 30 μl of the biomass were gently homogenized between glass coverslips. Following FISH (see earlier), coverslips were coated with Ilford K5D emulsion (Polysciences, Inc., Warrington, PA, United States), exposed in the dark for periods of 10 days and developed with Kodak D-19 developer.

### Raman Microspectroscopy

Raman spectra from single cells of *M. glycogenica* Lg2^T^ were obtained using a Horiba LabRam HR 800 Evolution (Jobin Yvon, France) equipped with a Torus MPC 3000 (United Kingdom) 532 nm 341 mW solid-state semiconductor laser. The incident laser power density on the sample was attenuated down to 2.1 mW/μm^2^ using a set of neutral density (ND) filters. The Raman system is equipped with an in-built Olympus (model BX-41) fluorescence microscope. A 50×, 0.75 numerical aperture dry objective (Olympus M-Plan Achromat, Japan), with a working distance of 0.38 mm was used throughout the work. A diffraction grating of 600 mm/groove was used, and the Raman spectra collected spanned the wavenumber region of 200 cm^-1^ to 1800 cm^-1^. The slit width of the Raman spectrometer and the confocal pinhole diameter were set to 100 and 150 μm, respectively. Raman spectrometer operation and subsequent processing of spectra were conducted using LabSpec version 6.4 software (Horiba Scientific, France). *Micropruina* cells from the pure cultures were directly mounted on optically polished CaF_2_ Raman windows (Crystran, United Kingdom) and air-dried before analysis. Spectra presented are average measurements from 30 individual *Micropruina* cells. Prior to all measurements, the Raman spectrometer was calibrated based on the first-order Raman signal of silicon, occurring at 520.7 cm^-1^. The CaF_2_ Raman substrate also contains a single-sharp Raman marker at 321 cm^-1^, which serves as an internal reference point in every spectrum. Glycogen (sourced from oyster – CAS No. 9005-79-2) (Sigma–Aldrich, United Kingdom) was used as reference compound. Glycogen produces characteristically strong Raman markers between wavenumbers 478–484 cm^-1^ and 840–860 cm^-1^, attributed, respectively, to skeletal deformation and CC skeletal stretch (Lin-Vien et al., 1991). PHA produces characteristic Raman bands at 432, 840, and 1726 cm^-1^, attributed, respectively, to δ (CC) skeletal deformations and ν(C=O) stretching vibrations (Lin-Vien et al., 1991; Supplementary Figure S1). Polyphosphate produces marker Raman bands at 1170 and 690 cm^-1^, attributed, respectively, to –P–O–P– stretching vibrations and ${\text{PO}}_{2}^{-}$ stretching vibrations (Majed et al., 2009; Supplementary Figure S1).

### FISH–Raman Analysis of *Micropruina*

In order to measure storage compounds (glycogen, PHA, and polyphosphate) *in situ* for the *Micropruina* spp., Raman microspectroscopy was combined with FISH. Activated sludge samples from the Ejby Mølle WWTP were incubated anaerobically with either glucose or butyrate (see MAR incubation details) and PFA fixed. FISH was performed on calcium fluoride slides using the MGL-67 and EUBmix probes. FISH-positive cells were located under fluorescence and, before Raman measurements, fluorescent labels were bleached by constantly illuminating the Raman laser on the area of interest for 5 min. Raman spectra were obtained from 30 single cells tagged with the *Micropruina* probe, as described above, and the average spectrum was calculated.

## Results and Discussion

### Pure Culture Physiology of *M. glycogenica*

*Micropruina glycogenica* Lg2^T^ is the sole isolate of the genus and its basic characterization has been reported previously (Shintani et al., 2000). The isolate was reportedly able to accumulate large amounts of an intracellular carbohydrate (up to 8.4% dry cell weight) under both aerobic and anaerobic conditions. The ability for *M. glycogenica* to grow under anaerobic conditions was not explicitly stated in the original description of the organism and neither the identity of the storage compound, nor the source of energy required for its storage under anaerobic conditions, have been determined. In the current study, anaerobic growth was confirmed with visible biomass in liquid culture observed after 7 days in R2A media (with no starch). Metabolite analyses with NMR suggested that, under anaerobic conditions, *M. glycogenica* primarily utilized available glucose and alanine as the primary carbon sources. Smaller amounts of trehalose (all present in the media was utilized), and the amino acids aspartate, glycine, histidine, leucine, and threonine were also detectably taken up from the media. Large amounts of acetate and lactate accumulated in the media, along with smaller fractions of ethanol and propionate, indicating active fermentation (Supplementary Figure S2). All of these by-products are reportedly utilized by at least one known PAO and GAO *in situ* (Kong et al., 2004, 2006; Burow et al., 2007; Wong and Liu, 2007; Nguyen et al., 2012). Raman microspectroscopy identified the storage polymer glycogen, but not PHA or polyphosphate, in anaerobically grown *M. glycogenica* cells (Supplementary Figure S3).

### Genetic Potential of *M. glycogenica*

Genome sequencing and assembly using Illumina short reads and Nanopore long reads for scaffolding gave a closed 3.8 Mbp circular chromosome for *M. glycogenica* Lg2^T^ (see **Table 2** for details). The genome contained genes for a complete TCA cycle, a cytochrome *c* oxidase, and other electron transport chain complexes, supporting an ability for aerobic respiration. A respiratory nitrate reductase (narG: MPLG2_2671) along with a putative copper-containing nitrite reductase (nirK: MPLG2_2662) were also annotated, despite the organism reportedly being able to reduce nitrate to nitrite but no further (Shintani et al., 2000). The Lg2 genome also contained complete pathways for glycolysis, the pentose phosphate pathway, and glycogen synthesis. The absence of an annotated PHA synthase indicates an inability to form PHAs, supporting the Raman analyses.

**Table 2:** Genome properties of the closed *Micropruina glycogenica* Lg2^T^ genome.

Property	
Size	3.84 Mbp
GC content	68.3%
Protein coding density	90.2%
CDS	3952
CDS assigned function^∗^	15.4%
rRNA operons	1
Sequencing project Accession No.	PRJEB23532

*CDS, coding DNA sequence. ^∗^MicroScope software prediction classes 1–3.*

Several annotated genes support the fermentative physiology observed with pure culture investigations (see earlier). Alanine and glucose, the main carbon sources utilized in pure culture incubations, are likely converted to pyruvate via the Embden–Meyerhof–Parnas (EMP) glycolysis pathway and by annotated alanine dehydrogenases (MPLG2_3627; MPLG2_3728), respectively. Pyruvate can be converted to acetyl-CoA by a pyruvate: ferredoxin oxidoreductase (MPLG2_2718), pyruvate dehydrogenase (MPLG2_1287; MPLG2_2466), or a pyruvate formate lyase (MPLG2_2587), with formate released from activity of the latter potentially oxidized to CO_2_ by an annotated formate dehydrogenase (MPLG2_0499-0500). Fermentation by-products from acetyl-CoA include acetate, mediated by a phosphate acetyltransferase (MPLG2_3027) and an acetate kinase (MPLG2_v2_2721) and ethanol, facilitated by a possible acetaldehyde dehydrogenase (MPLG2_v2_0028) and an alcohol dehydrogenase (MPLG2_0623). Detected lactate was likely generated from pyruvate, catalyzed by an annotated lactate dehydrogenase (MPLG2_0671). Key genes were missing from the acrylate pathway (Cardon and Barker, 1947) ruling it out as the source of observed propionate accumulation under anaerobic conditions. The small amount of propionate produced may come from the metabolism of some amino acids and/or the anaerobic activity of the TCA cycle in conjunction with the methylmalonyl-CoA pathway – similar to proposed pathways for the production of propionyl-CoA in the classical GAO phenotype (Mino et al., 1998).

### Abundance and Distribution of *Micropruina* spp. in Full-Scale Systems

**FIGURE 2:**
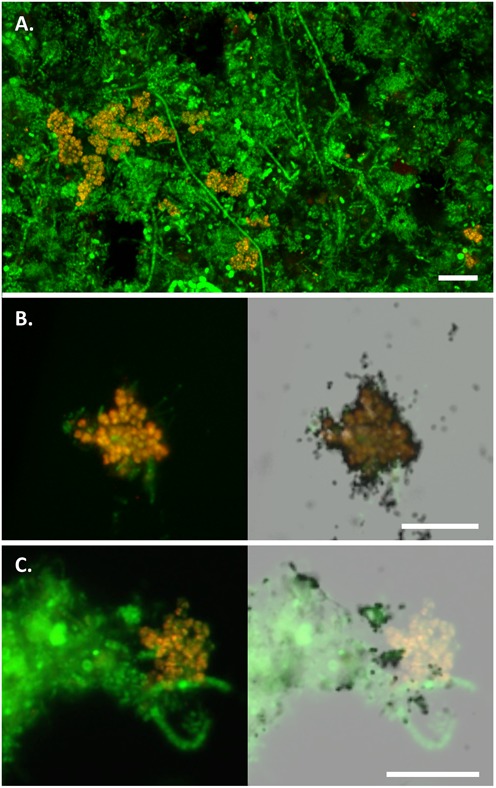
FISH micrographs of the *Micropruina* spp. in full-scale activated sludge. In all images, *Micropruina* appear yellow [MGL-67 (red) + EUBmix (green)] and other bacteria green (EUBmix alone). **(A)** Composite fluorescence image of the Ejby Mølle WWTP sludge. **(B,C)** MAR-FISH image sets: composite fluorescence image alone (left) and with overlay of corresponding field of view with bright-field microscopy showing MAR signal (right). Cells with associated silver granules (black dots) are positive for uptake of the labeled substrate; **(B)** positive anaerobic uptake of ^3^H-glucose; **(C)** negative aerobic uptake of ^3^H-acetate. Scale bars indicate 10 μm.

**FIGURE 3:**
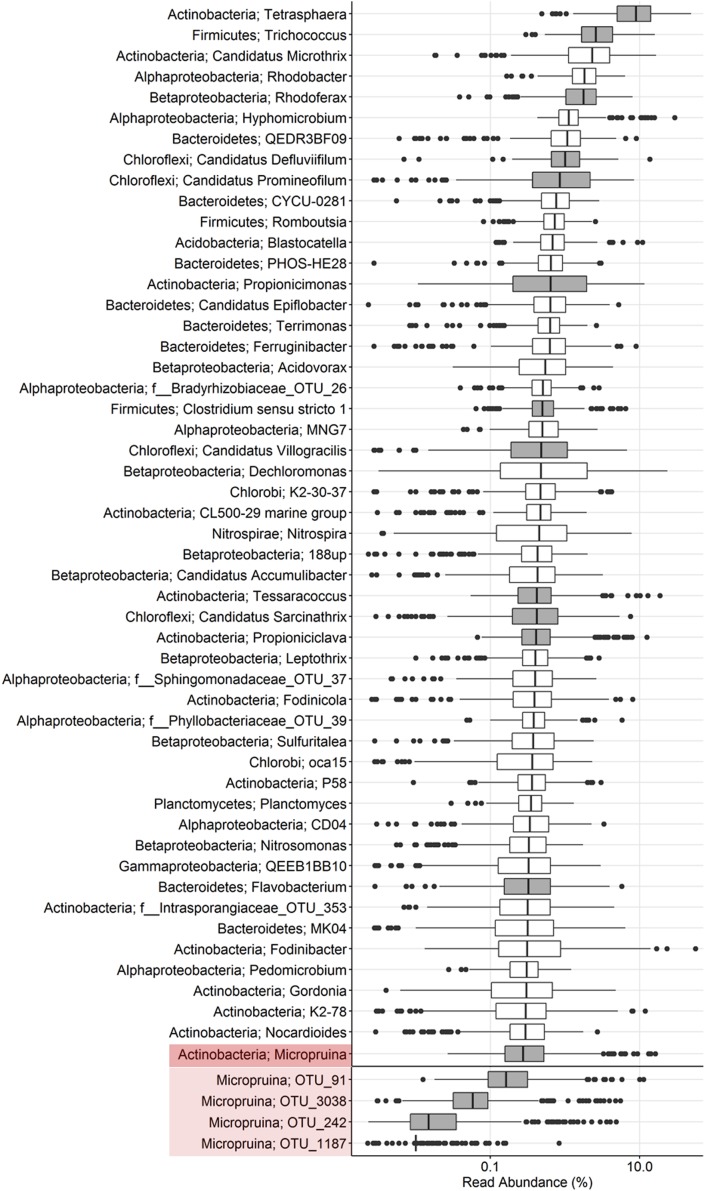
Boxplot showing the abundance distribution of the top genus-level phylotypes as well as individual OTUs belonging to the genus *Micropruina* in Danish EPBR plants. The data source is an extensive 16S rRNA gene amplicon sequencing (V1–3 region) survey of 18 plants over an 8-year period (2006–2014). For further details, see Stokholm-Bjerregaard et al. (2017). Phylotypes with gray box plots are those known to be associated with a fermentative metabolism.

In this study, two FISH probes were designed to target the *Micropruina* genus (**Table 1**). Both have the same coverage of the genus with *in silico* analyses and can be applied together with different fluorochromes to increase confidence in their specificity (**Table 1** and **Figure 1**). These probes replace the MIC184 probe (Kong et al., 2001), which has inferior specificity (**Table 1**). Pre-treatment with lysozyme was required to give good FISH signal for the *Micropruina* cells. Neither achromopeptidase nor mild acid treatment, which have been applied to increase permeability for other Gram-positives (?), led to a noticeable increase in signal. Cells responding to the MGL-67 and MGL-1223 FISH probes were cocci, typically between 0.5 and 1.0 μm in diameter, forming tetrads and microcolonies (**Figure 2**), which is consistent with the description of *M. glycogenica* Lg2^T^ (Shintani et al., 2000). Despite covering 90% of near full-length sequences assigned to the genus in the MiDAS database (**Table 1**), the probes of this study do not cover all members of the genus in full-scale systems, and their use may underestimate *Micropruina* abundance (see Supplementary Text). However, they do cover the most abundant member of the genus (represented by OTU_91: **Figures 1**, **3** and Supplementary Figures S4, S5).

Few other studies have investigated the abundance of the *Micropruina* in full-scale systems. They were observed in Japanese full-scale EBPR systems at up to 8% of the biovolume (Wong et al., 2005) but were absent from several plants surveyed in Australia (Beer et al., 2006). It should be noted that lysozyme pre-treatment – which we have shown in the current study to be important to obtain a good FISH signal for members of the genus – was not performed in either study. This may have led to their underestimation or lack of detection. Amplicon sequencing surveys suggest that they are abundant in WWTPs globally, and therefore likely important (Nierychlo and Nielsen, unpublished data).

### *In Situ* Physiology of the Abundant *Micropruina* spp.

Microautoradiography–fluorescence *in situ* hybridization analyses of activated sludge from two WWTPs showed that FISH-positive *Micropruina* cells were positive for uptake of a broad range of tested substrates, including glucose, fructose, galactose, amino acids, pyruvate, glycerol, and butyrate, under both aerobic and anaerobic conditions. NAG, ethanol, acetate, propionate, and oleate were not assimilated (Supplementary Table S1 and Supplementary Figures S6, S7). In contrast, *M. glycogenica* Lg2^T^ is reportedly able to utilize both acetate and propionate under aerobic conditions (Shintani et al., 2000). This may indicate a metabolic difference between the pure culture and *in situ* strains. However, it should be noted that activated sludge organisms are known to behave different in pure culture to when they are in mixed communities where they tend to be more specialized feeders (e.g., Kindaichi et al., 2013; McIlroy et al., 2013). The abundant members of the genus may not be able to competitively take up enough of these VFA substrates to give a positive signal with the MAR–FISH method. The uptake of sugars and amino acids is consistent with the fermentative metabolism observed for *M. glycogenica* Lg2^T^; however, future efforts to obtain genomes from *in situ* members of the genus will more definitively support a role for these organisms in fermentation in EBPR systems. The ability for the *in situ Micropruina* spp. to utilize sugars and amino acids is shared by the *Tetrasphaera* PAO (Kong et al., 2005), indicating direct competition for anaerobic carbon for these organisms. Raman microspectroscopy combined with FISH was applied to show that cells contained glycogen, but not PHAs, after anaerobic incubation with either glucose or butyrate (Supplementary Figure S3). MAR-FISH confirmed these substrates were incorporated into the *Micropruina* cells *in situ* – noting that the change in internal glycogen during the incubation would need to be quantified to empirically determine if supplied substrates are used for growth or storage. Cells from the aeration tank did not contain detectable polyphosphate. These results are consistent with the Raman results for *M. glycogenica* Lg2^T^ (see earlier). Anaerobic utilization of butyrate *in situ* is difficult to explain, noting that Raman–FISH analyses indicated that PHAs were not produced with butyrate uptake (data not shown). The pure culture did not utilize butyrate when supplemented into the R2A media (at 2 mM) – noting that a range of other carbon sources were available that may be utilized preferentially – and the Lg2^T^ genome lacks a butyrate kinase. It may be that *in situ* strains utilize butyrate for anabolic reactions, with stored glycogen as the primary source of carbon and energy. Further work is required to determine this.

### A Metabolic Model for the *Micropruina* in EBPR Systems

The *Micropruina* genus did not possess the classical GAO metabolism, but likely exhibits a fermentative metabolism in EBPR systems. Under anaerobic conditions these organisms are proposed to ferment sugars and amino acids to provide the carbon source and energy required for glycogen storage and possibly growth. As the *Micropruina* spp. are facultative anaerobes, stored glycogen may be oxidized under aerobic conditions for growth. Such a metabolic model makes them ideally suited to the dynamic feast–famine conditions of EBPR. Members of the genus enriched in a previous lab-scale study, reportedly able to assimilate acetate as PHA (Kong et al., 2001), may represent atypical members of the genus as neither the abundant *in situ* members of the genus, nor the axenic culture of *M. glycogenica*, behaved in this way. Therefore, the abundant *Micropruina* did not behave according to the classical GAO model where VFAs are stored as PHA under anaerobic conditions. They do accumulate glycogen, but under anaerobic conditions where excess substrate is available, and are therefore considered to behave as “fermentative GAO” (fGAO). Anaerobic glycogen accumulation has been reported for non-EBPR sludges previously (Liu et al., 1996; Carucci et al., 1997), that may have contained undescribed fermentative GAOs, as well as for the *Tetrasphaera* PAO isolates (Kristiansen et al., 2013).

### Implications for EBPR Research

**FIGURE 4:**
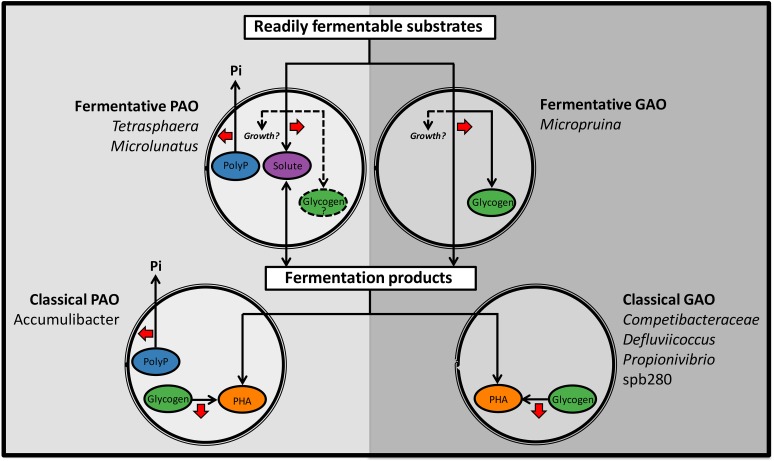
Schematic diagram summarizing the important anaerobic transformations for known phenotypes with suggested importance to EBPR. Abundant phylotypes are included for each phenotype. Phenotypes to the left (PAO) favor EPBR and those to the right (GAO) are suggested to be detrimental to the process. Red arrows indicate net energy generation. In brief, the fermentative PAO (fPAO) compete with the fGAO and other fermenters for fermentable substrates (such as sugars and amino acids) and the classical PAO and GAO compete for fermentation by-products (such as VFAs). The *Micropruina* spp., and other potential fGAO, store some carbon as glycogen. The *Tetrasphaera* spp. and *Microlunatus* fPAO reportedly store some carbon as non-polymerized fermentation by-products and have the potential for glycogen storage (Santos et al., 1999; Kristiansen et al., 2013; Nguyen et al., 2015; Nguyen, H., and Nielsen, P.H., unpublished data).

In light of the findings of the current study, competition for resources likely occurs on two levels, where the fermentative PAO (fPAO) compete with the fGAO and other fermenters for sugars and amino acids, and the classical PAO and GAO compete for fermentation by-products such as VFAs (summarized in **Figure 4**). Interestingly, the ability of the fPAO and fGAO to grow under both aerobic and anaerobic conditions may provide them with an advantage over the organisms with the classical PAO and GAO phenotypes, which is at least consistent with their higher relative abundances in Danish WWTPs (Stokholm-Bjerregaard et al., 2017). In addition, while the *Micropruina* spp., and other unknown fGAO, may ferment substrates to supply VFAs, any carbon stored as glycogen and utilized aerobically is not made available anaerobically to the classical PAOs for subsequent P removal – which may be important in systems where VFAs are limiting. As such, organisms storing carbon under anaerobic conditions, in the absence of polyphosphate cycling, are potential competitors of the PAO.

The dynamic feast–famine EBPR environment is selective for organisms able to store carbon anaerobically. Fermenters storing carbon when it is in excess will be able to use it for growth under aerobic conditions, but also during any anaerobic starvation periods (van Loosdrecht et al., 1997). Supporting the anaerobic storage of carbon by fermenters, Kong et al. (2008) observed the continual accumulation of VFAs with anaerobic incubation of full-scale sludge. This occurred when the carbon storage capacity of the classical PAO and GAO was saturated and added exogenous carbon sources became exhausted, which likely indicates fermentation of storage products by unidentified bacteria. In full-scale BNR plants, *in situ* measures estimate that bacteria identified to be actively fermentative constitute up to 40% of the biovolume and appear to be largely refined to the Actinobacteria and Firmicutes (Kong et al., 2008; Nielsen et al., 2012). Previous *in situ* studies have identified the genera *Propionicimonas*, *Tetrasphaera*, and “*Ca.* Promineofilum” (Chloroflexi) as abundant fermenters in full-scale systems (Kong et al., 2008; Nielsen et al., 2012; McIlroy et al., 2016). These genera, along with several others known to include fermentative species, are among the most abundant in comprehensive surveys of full-scale systems in Denmark – in even higher abundances than *Micropruina* (**Figure 3**). Future application of Raman–FISH, or equivalent methods, to quantify the storage polymer dynamics of abundant organisms, will provide important information regarding the flow of carbon through these systems and the potential impact of specific abundant organisms on P removal and the ecology of EBPR. Elucidating the physiology of these abundant members of the community will importantly contribute to the broader goal of an in-depth understanding of the ecology of EBPR systems. To this end, the current study provides a detailed insight into the ecology of the abundant *Micropruina* spp., with the designed FISH probes and genome providing the foundation for more detailed studies – including *in situ* storage polymer dynamics and gene expression studies.

## Author Contributions

SM, PN, F-AH, and CO planned the experiments. F-AH, CO, and BM performed the pure culture incubations and F-AH carried out the NMR analyses with help from RW. CO and JK prepared the genomic DNA. MD, RK, and SK sequenced and assembled the genome, and SM annotated the genome. FISH probe design, optimization, and quantitative FISH analyses were performed by SM and BM. CO, EF, and MN performed the single cell analyses. The manuscript was drafted by SM and revised by all the authors.

## Conflict of Interest Statement

The authors declare that the research was conducted in the absence of any commercial or financial relationships that could be construed as a potential conflict of interest.
