# Supplementary material for: Genomic and in Situ Analyses Reveal the Micropruina spp. as Abundant Fermentative Glycogen Accumulating Organisms in Enhanced Biological Phosphorus Removal Systems
Source: Front Microbiol. 2018 May 23;9:1004. doi: 10.3389/fmicb.2018.01004 (PMC5974061; doi:10.3389/fmicb.2018.01004)
Supplement: Supplementary file 1 [file Presentation_1.PDF]

SUPPLEMENTARY INFORMATION

Genomic and *in situ* analyses reveal the *Micropruina* spp. as abundant fermentative glycogen accumulating organisms in enhanced biological phosphorus removal systems

Simon J. McIlroy, Cristobal A. Onetto, Bianca McIlroy, Florian-Alexander Herbst, Morten Simonsen Dueholm, Rasmus H. Kirkegaard, Eustace Fernando, Søren M. Karst, Marta Nierychlo, Jannie M. Kristensen, Kathryn L. Eales, Paul R. Grbin, Reinhard Wimmer and Per Halkjær Nielsen

FISH probe coverage analysis

Large scale surveys of full-scale activated sludge plants in Denmark revealed a relatively high abundance of the *Micropruina* spp. (Stokholm-Bjerregaard *et al.*, 2017). Quantitative FISH (qFISH) was applied in the current study to verify these abundances. Relatively high abundances of the genus were observed with qFISH analyses, where they constituted up to 6% of the biovolume, although lower values were determined relative to the amplicon read data for the same full-scale samples (**Figure S4**). This is explained by analyses of the abundant OTU sequences of the MiDAS survey (McIlroy *et al.*, 2015). There are three abundant OTUs and the MGL-67 probe target site is found in OTU-95 but not in the less frequently observed OTU-239 and OTU-3062 (**Figure 1, 3 & S4**). The latter two OTUs are abundant in the Randers and Hirtshals WWTPs, where a large difference was observed between the qFISH and amplicon abundance estimations (**Figure S4**). Coverage of the MGL-1223 probe could not be assessed, as the amplicons do not contain sequence information for the site. However good overlap for the MGL-67 and MGL-1223 probes was observed in all plants assessed in this study except Hirtshals (**Figure S5**), indicating that the latter probe does not cover these species either. The qFISH abundances correspond well with OTU-91 read abundance indicating that quantification with the amplicon method provides a good estimate of genus abundance. Therefore, the probes of this study appear to cover the abundant member of the genus, but not all members in full-scale systems, and their general use may underestimate *Micropruina* abundance.

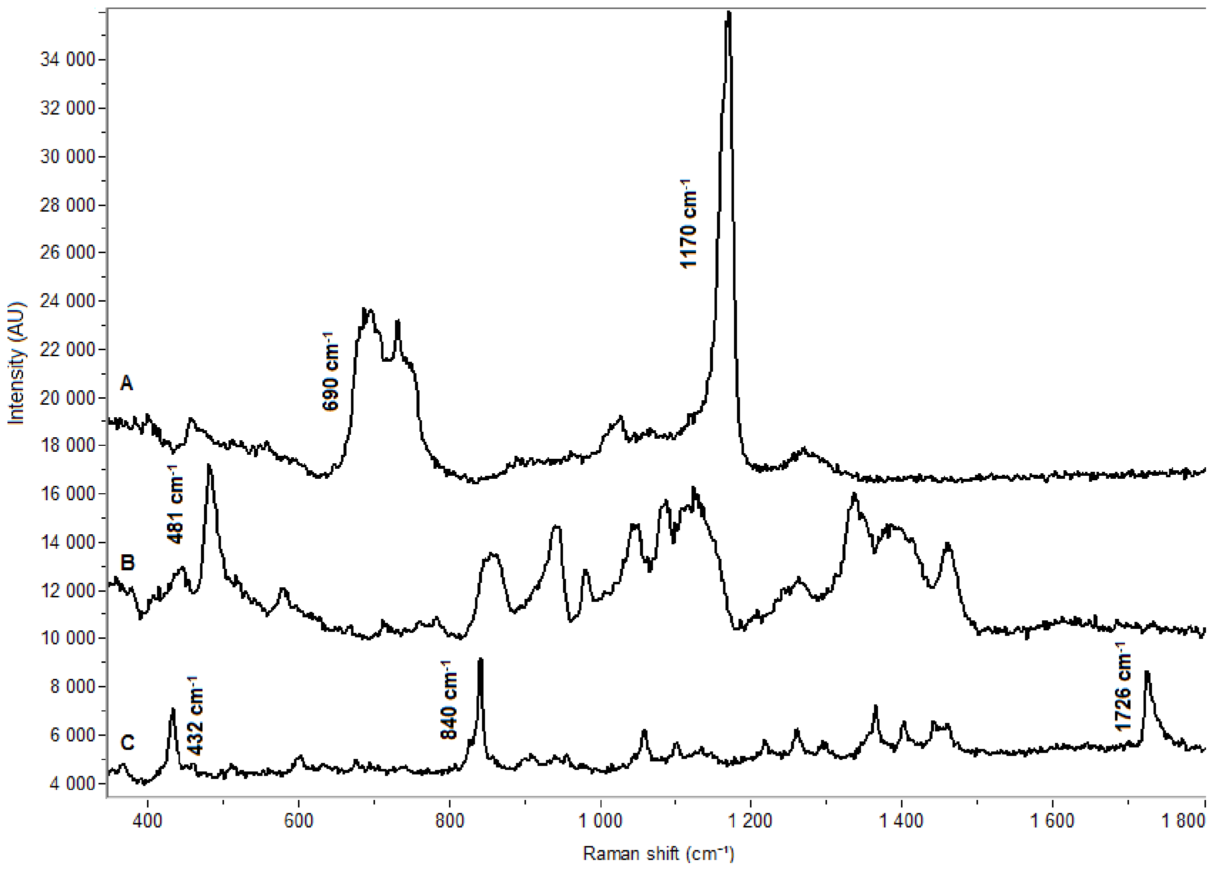

Figure S1. Raman spectra overlay of (A) polyphosphate (B) glycogen and (C) poly (3-hydroxybutyric acid-co-3-hydroxyvaleric acid) standard compounds indicating their characteristic Raman marker peak positions.

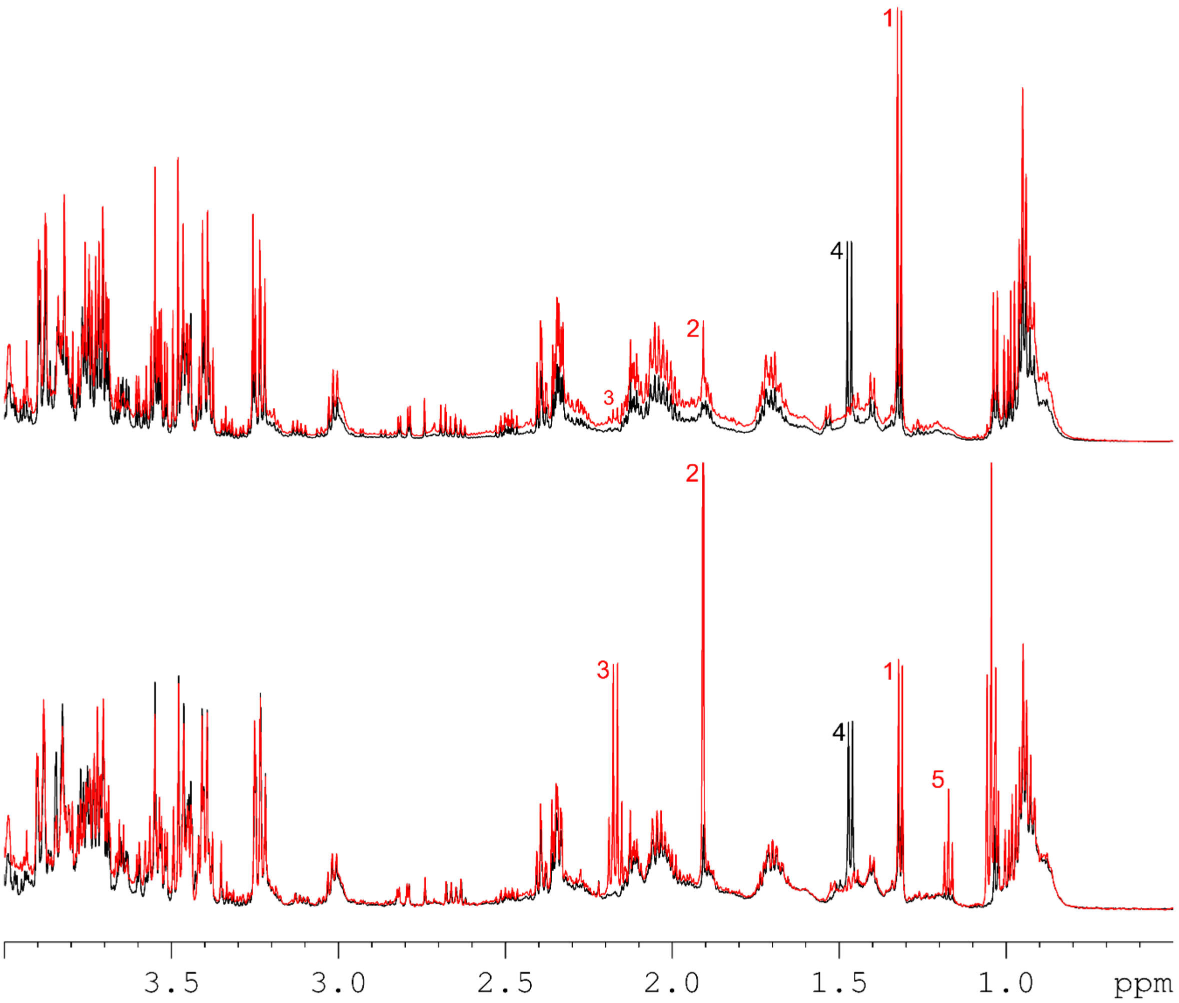

Figure S2. Example of NMR spectra for metabolite quantification. Original spectra range from 10 to -1 ppm and contain additional peaks for identification as well as quantification. One out of four biological replicates are chosen and shown before (black) and after cultivation (red) with (top) and without an additional concentration step (bottom). Numbers identify signature peaks for lactate (1), acetate (2), propionate (3), alanine (4), and ethanol (5). Example plots were created in Bruker TopSpin (v.3.5).

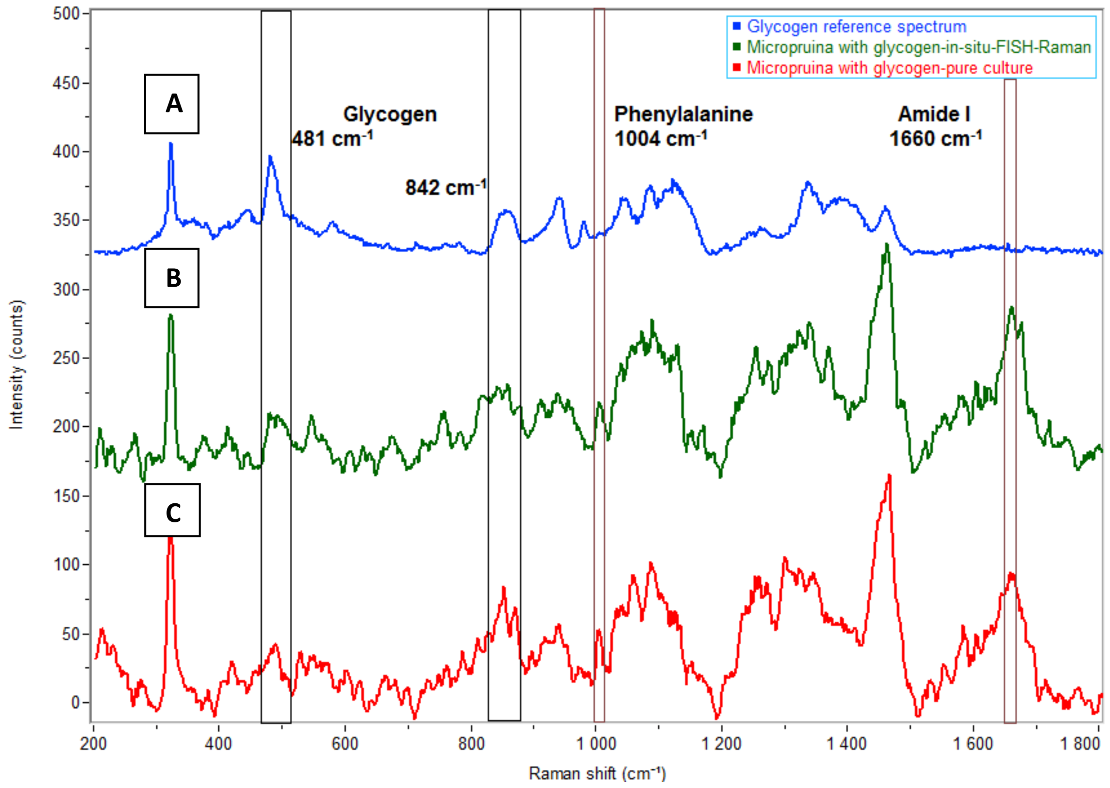

Figure S3. Overlay of the Raman spectra for (A) pure glycogen (B) *Micropruina* spp. FISH probe (MGL-67) and (C) *M. glycogenica* Lg2^{T} pure culture labelled cells indicating the characteristic Raman marker for glycogen at 481 cm^{-1}.

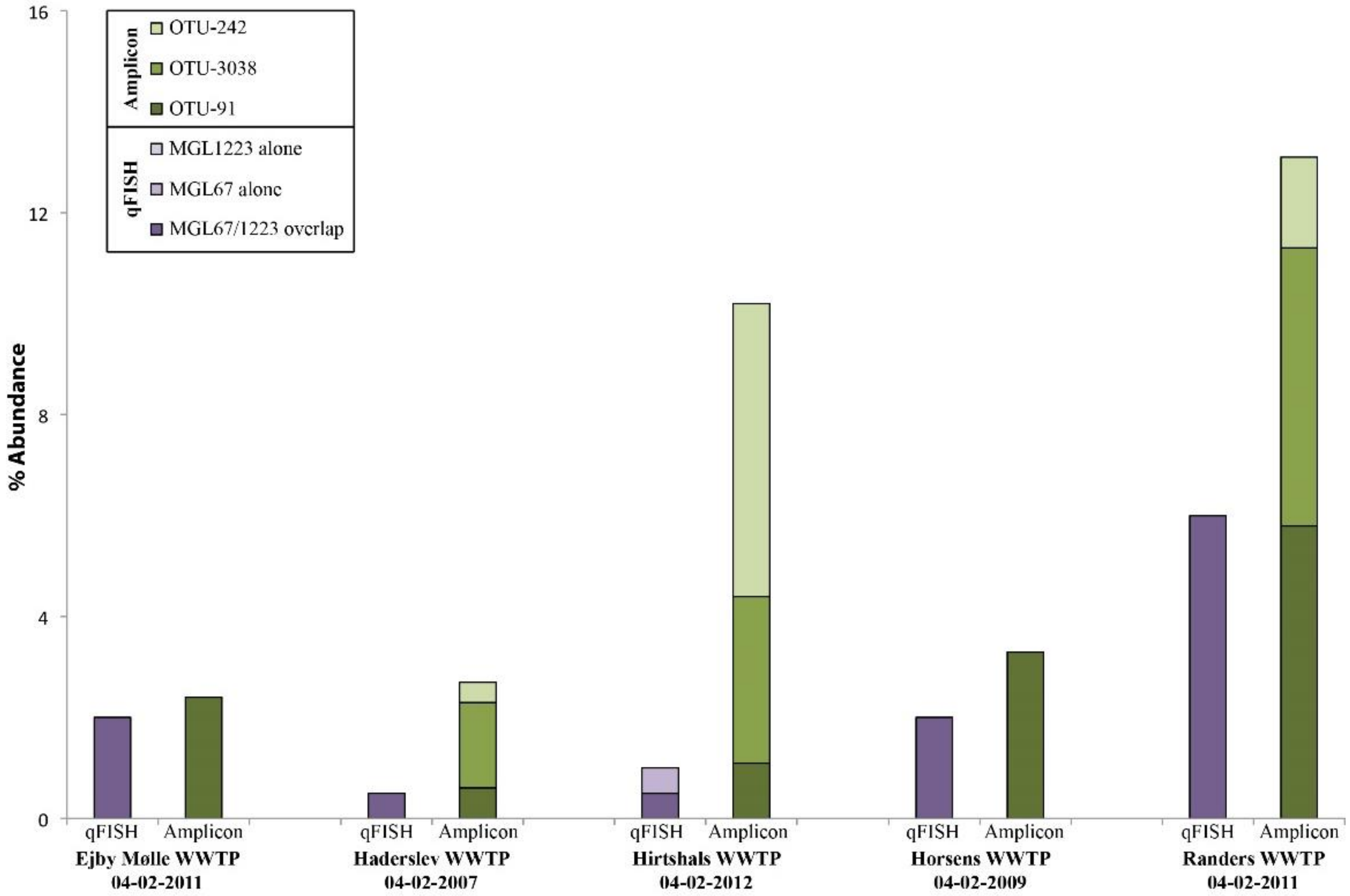

Figure S4. Comparison of FISH and 16S rRNA gene amplicon sequencing for estimating the relative abundance of *Micropruina* spp. in full-scale systems. Amplicon sequencing data is sourced from the MiDAS survey (McIlroy *et al.*, 2015).

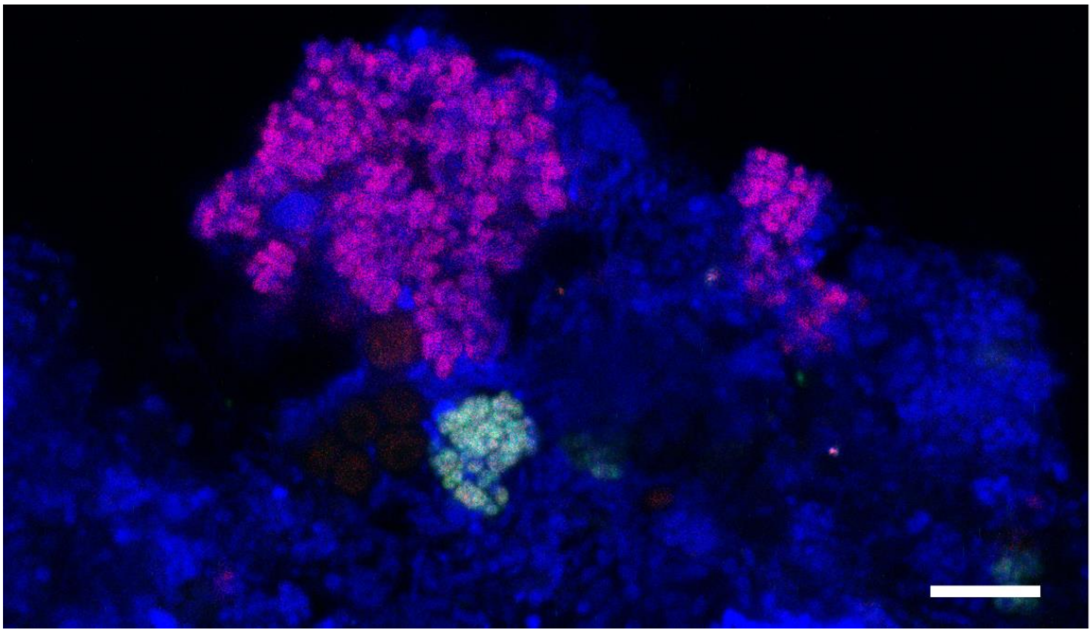

Figure S5. Composite FISH micrograph of biomass from the Hirtshals WWTP. Cells with a positive signal for both *Micropruina* spp. probes appear white (MGL-67 (green) + MGL-1223 (red) and EUBmix (blue)), cells hybridising only MGL-1223 appear magenta (MGL-1223 (red) + EUBmix (blue)) and those hybridising the EUBmix probe set only appear blue. Scale bar represents 10 µm.

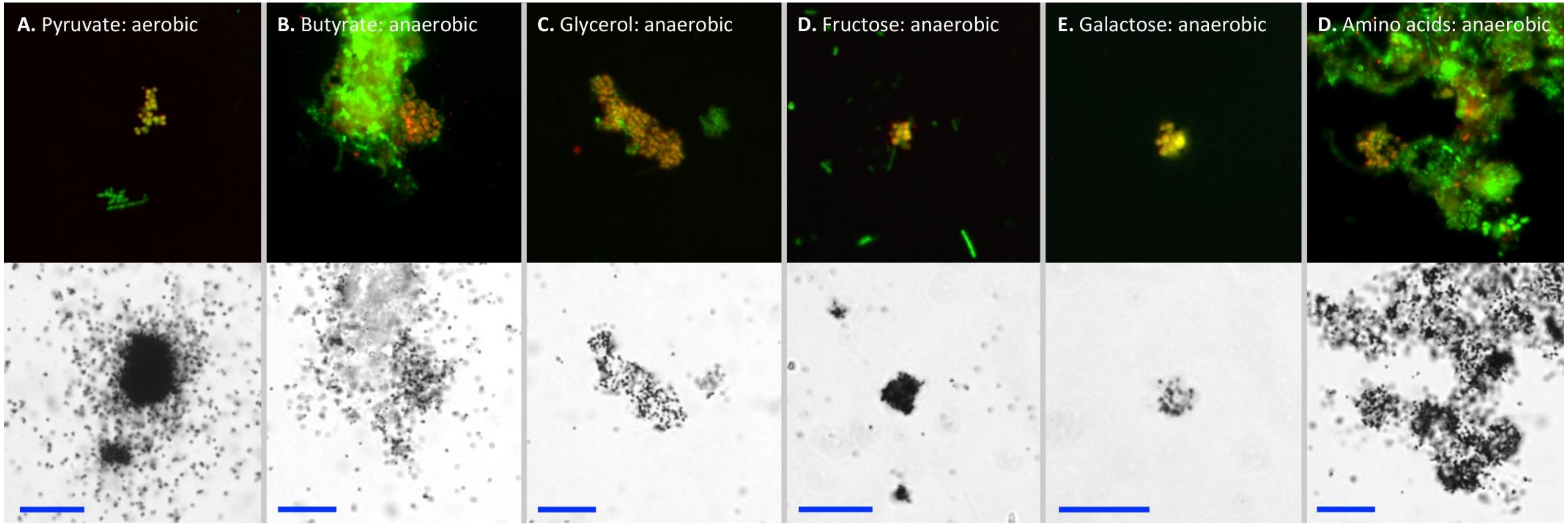

Figure S6. FISH and corresponding bright-field MAR micrographs depicting positive carbon source uptake for *Micropruina* spp. in sludge from full-scale WWTPs. Labelled substrates and electron acceptor conditions are indicated for each image. *Micropruina* spp. cells in FISH micrograph overlays appear yellow (MGL-67 (red) + EUBmix (green) and non-target green (EUBmix only). Black silver granules indicate positive MAR signal. Scale bars represent 10 µm. Results are summarized in **Table S1**.

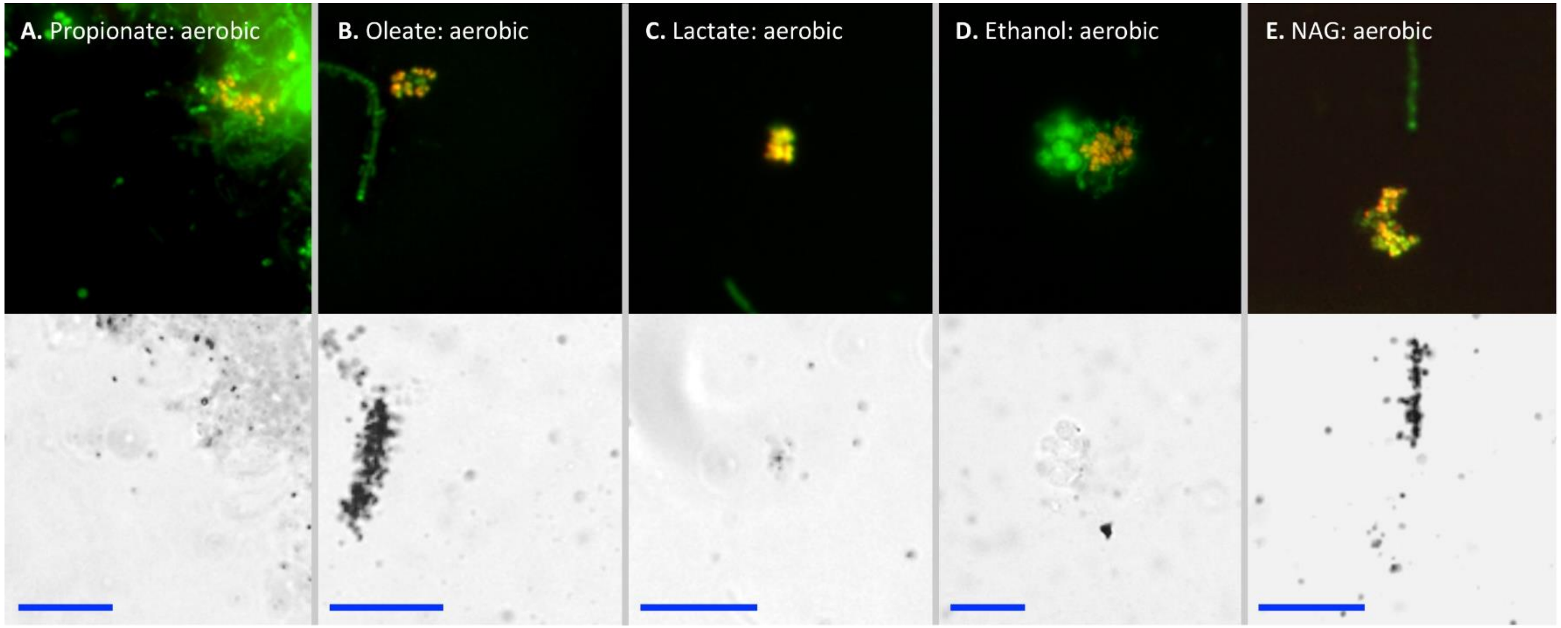

Figure S7. FISH and corresponding bright-field MAR micrographs depicting negative carbon source uptake for *Micropruina* spp. in sludge from full-scale WWTPs. Labelled substrates and electron acceptor conditions are indicated for each image. *Micropruina* spp. cells in FISH micrograph overlays appear yellow (MGL-67 (red) + EUBmix (green) and non-target green (EUBmix only). Black silver granules indicate positive MAR signal. Scale bars represent 10 µm. Results are summarized in **Table S1**.

Table S1: Summary of MAR-FISH results for carbon uptake profiles.

Substrate	Aerobic	Anaerobic
Glucose	++	++
Fructose	++[1]	++[1]
Galactose	++[1]	++[1]
NAG	−	NA
Amino acids	++	+[2]
Pyruvate	++[2]	NA
Glycerol	++	++[2]
Ethanol	−	NA
Acetate	−	–[2]
Propionate	−	NA
Butyrate	+	++[2]
Oleate	–[2]	NA

Data represents the proportions of 20 FISH-positive cells that are also MAR positive: −, No MAR positive cells; +, 60-80%; ++, > 80%. NA= Not assessed. **1.** Only determined for species in the Odense NW WWTP. **2.** Only determined for species in the Ejby Mølle WWTP.

REFERENCES

McIlroy SJ, Saunders AM, Albertsen M, Nierychlo M, McIlroy B, Hansen AA, *et al.* (2015). MiDAS: the field guide to the microbes of activated sludge. *Database* **2015**: 1–8.

Stokholm-Bjerregaard M, McIlroy SJ, Nierychlo M, Karst SM, Albertsen M, Nielsen PH. (2017). A Critical Assessment of the Microorganisms Proposed to be Important to Enhanced Biological Phosphorus Removal in Full-Scale Wastewater Treatment Systems. *Front Microbiol* **8**: 718.
